# Morphological, Release and Antibacterial Performances of Amoxicillin-Loaded Cellulose Aerogels

**DOI:** 10.3390/molecules23082082

**Published:** 2018-08-20

**Authors:** Shan Ye, Shu He, Chen Su, Lei Jiang, Yanyi Wen, Zhongjie Zhu, Wei Shao

**Affiliations:** 1 College of Chemical Engineering, Nanjing Forestry University, Nanjing 210037, China; 13451867328@163.com (S.Y.); heshu999@163.com (S.H.); 13260866882@163.com (C.S.); 15655679696@163.com (L.J.); wenyanyis@163.com (Y.W.); 13382367651@163.com (Z.Z.); 2 Jiangsu Key Lab for the Chemistry & Utilization for Agricultural and Forest Biomass, Nanjing Forestry University, Nanjing 210037, China

**Keywords:** cellulose, amoxicillin, controlled release, antibacterial

## Abstract

Cellulose has been widely used in the biomedical field. In this study, novel cellulose aerogels were firstly prepared in a NaOH-based solvent system by a facile casting method. Then amoxicillin was successfully loaded into cellulose aerogels with different loadings. The morphology and structure of the cellulose aerogels were characterized using scanning electron microscopy (SEM) and Fourier transform infrared spectroscopy (FTIR). The drug release and antibacterial activities were also evaluated. The drug release results showed that cellulose aerogels have controlled amoxicillin release performance. In vitro antibacterial assay demonstrated that the cellulose aerogels exhibited excellent antibacterial activity with the amoxicillin dose-dependent activity. Therefore, the developed cellulose aerogels display controlled release behavior and efficient antibacterial performance, thus confirming their potential for biomedical applications.

## 1. Introduction

Aerogels are three-dimensional network materials derived from gel networks by replacing the liquid medium with gas [1]. They show great promise for using as supporting substrate, owing to their high surface area, low density, high specific surface area, recyclability, biodegradability, low cost and aqueous stability [2]. Therefore, aerogels have a variety of applications, such as acoustic insulation, CO_2_ capture, catalyst supports, drug carrier and membrane separations [3,4,5,6].

Cellulose is the most abundant natural renewable polymer on Earth. It is a linear polysaccharide formed by β-1-4-linked d-glucopyranose repeating units [7]. It has gained special attention due to its biodegradability, hydrophilicity, biocompatibility, non-toxicity, high mechanical strength, and thermal and chemical stability [8]. Cellulose has potential in various applications, such as the paper industry, medicine and electronics industry, packaging materials, energy areas, hygiene and biomedical industries, and the cosmetic and food industries [9,10]. In recent years, cellulose aerogels have received increasing research interest in biomedical fields concerning tissue engineering, controllable delivery systems, and wound dressings [11]. However, cellulose has no inherent antibacterial capacity, resulting in the limitation of applications in some medical areas. One simple solution is to load antibacterial agents into the cellulose aerogels. Silver nanoparticles have been reported to load into cellulose by many researchers to endow it with antibacterial and antifungal properties [12,13,14,15]. Cellulose and keratin composite-loaded gold nanoparticles were prepared and they exhibited good biocompatible and bactericidal capabilities [16]. Nisin-loaded oxidized cellulose was developed in a facile and green process with long-term antimicrobial active, which can be used as a safe and biodegradable material for food packaging [17]. A zinc oxide nanorod cluster-deposited cellulose sheet with remarkable antibacterial activity towards Gram-positive and Gram-negative bacteria was prepared and it has potential applications in pharmaceutical, biomedical, food packaging, water treatment and biotechnological industries [18].

Amoxicillin is a semisynthetic, β-lactam antibiotic with broad-spectrum antibacterial activity. It can inhibit the carboxypeptidase and transpeptidase enzymes which involves peptidoglycan biosynthesis [19,20]. An amoxicillin-loaded bone ash-reinforced chitosan-based hydrogel with pH-sensitivity was prepared with efficient and controlled release behavior [21]. The PEGylated PLGA was applied to load amoxicillin for subsequent formation of electrospun amoxicillin /PLGA-PEG nanofibers and the obtained nanofibers showed good antibacterial activity and cytocompatibility [19]. Amoxicillin loaded cysteine conjugated chitosan/PMLA multifunctional nanoparticles were designed and successfully fabricated, and they can be used as new promising oral drug delivery systems targeting *Helicobacter pylori* to increase local drug bioavailability [22].

Therefore, regenerated cellulose aerogels loaded with amoxicillin as controllable antibiotic delivery systems was fabricated in this study. The as-prepared aerogels were characterized by different techniques, such as scanning electron microscopy (SEM), Fourier transform infrared spectroscopy (FTIR) and thermogravimetric analysis (TG). The drug release performance of the obtained cellulose aerogels was studied. The antibacterial activity of the aerogels was also studied against Gram-negative bacteria *Escherichia coli* ATCC 25922 (*E. coli*), fungus *Candida albicans* CMCC(F) 98001 (*C. albicans*), Gram-positive bacteria *Staphylococcus aureus* ATCC 6538 (*S. aureus*) and *Bacillus subtilis* ATCC 9372 (*B. subtilis*).

## 2. Results and Discussion

### 2.1. Morphology 

**Figure 1:**
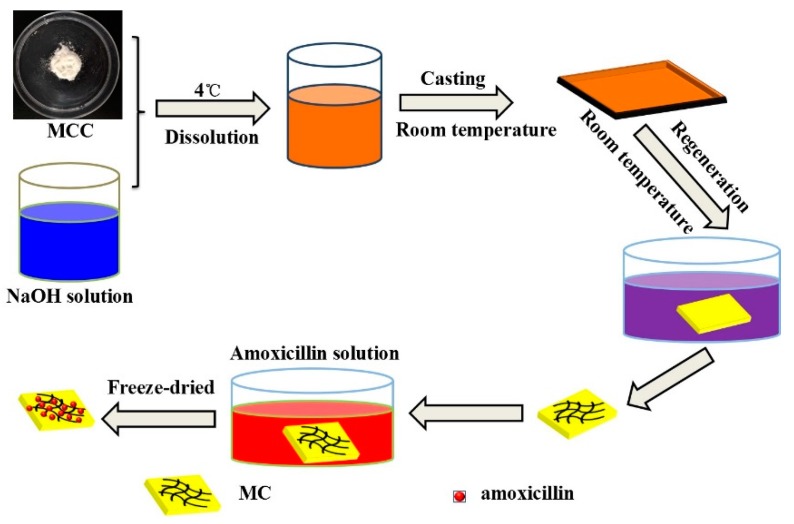
Schematic illustration of preparing cellulose aerogels.

The dissolution and regeneration processes of cellulose are on account of the disruption and formation of hydrogen, respectively. NaOH can penetrate not only between crystallites but also into the crystallites to destroy inter- and intra-hydrogen bonds between cellulose molecules [23]. Firstly, MC was dissolved by NaOH aqueous solution to form homogeneous cellulose solution. When the formed cellulose solution was immersed into a de-ionized water (non-solvent), NaOH molecules bonded on the –OH groups of cellulose diffused into water, so the new hydrogen bonds were regenerated. The rearrangement of the hydrogen bonds leads to the regeneration of cellulose. Due to the phase separation between solvent and non-solvent, many pores generate in the cellulose hydrogel, forming a three-dimensional network structure [24]. When cellulose hydrogel is immersed into amoxicillin solution, amoxicillin can be loaded onto cellulose fibers through the internal pores. The detailed preparation method of cellulose aerogels is shown in Figure 1.

**Figure 2:**
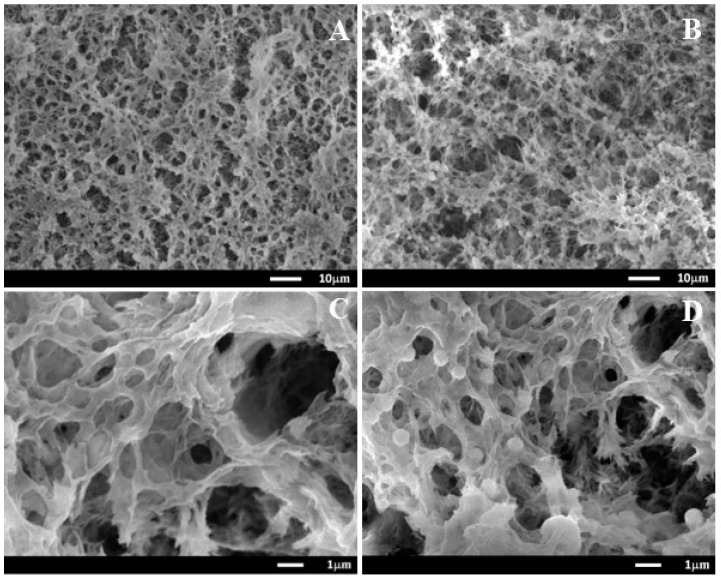
SEM images of MC (**A**,**C**) and MC_4_ (**B**,**D**) aerogels (**A**,**B**: ×1000, **C**,**D**: ×8000).

The morphologies of cellulose aerogels were analyzed using SEM (Figure 2). Figure 2A,C shows the morphology of MC aerogel, which exhibits a porous three-dimensional network structure. The microfibrils present a random arrangement without any preferential orientation, possessing a large surface area and high porosity, which favors drug loading. In the case of MC_4_ aerogel, amoxicillin particles were displayed as white spots distributed uniformly on the cellulose fibers (Figure 2B,D).

### 2.2. FTIR Characterization

**Figure 3:**
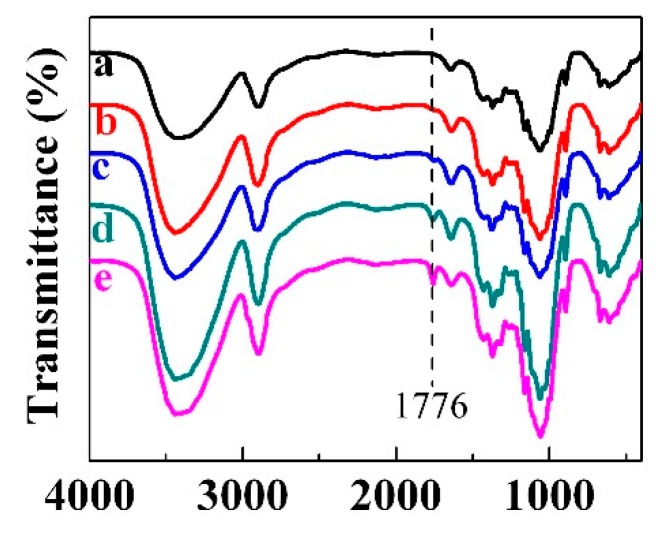
Fourier transform infrared (FTIR) spectra of MC (a), MC_1_ (b), MC_2_ (c), MC_3_ (d) and MC_4_ (e) aerogels.

FTIR spectra of cellulose aerogels with different loadings of amoxicillin are shown in Figure 3. For MC (curve a), the broad absorption band located from 3200 to 3700 cm^−1^ corresponds to the intramolecular hydrogen bond and the hydroxyl group. The peak at 2900 cm^−1^ is due to the existence of CH_2_ groups [25]. An absorption band at 1649 cm^−1^ is assigned to the bending vibrations of the primary and secondary O–H groups of cellulose [26]. Besides, the peaks at 1163, and 1061 cm^−1^ correspond to the C–O asymmetric bridge stretching and the C–O–C pyranose ring skeletal vibration, respectively [27]. In the case of amoxicillin-loaded MC aerogels (curve b–c), a new peak appears at 1776 cm^−1^, which is attributed to β lactam C=O stretching of amoxicillin [28]. Moreover, the peak intensity is highly enhanced with the increase of amoxicillin loading in the cellulose aerogel. The other characteristic peaks of amoxicillin are not obvious in the FTIR spectra, probably because they are merged into the large peaks of cellulose.

### 2.3. Thermogravimetric Analysis (TG) Analysis

**Figure 4:**
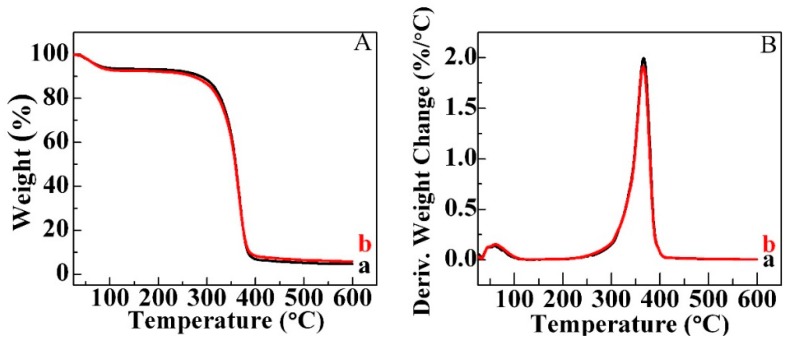
TG (**A**) and DTG (**B**) analysis of MC (a) and MC_4_ (b) aerogels.

TG is a continuous process, involving the measurement of sample weight in accordance with increasing temperature in the form of programmed heating. The thermal degradation behaviors of MC and MC_4_ aerogels were studied in the range of 25–600 °C under a nitrogen atmosphere. The TG and Derivative Thermo-gravimetry (DTG) spectra are shown in Figure 4A,B, respectively. There are two significant weight loss stages below 600 °C in the TG curve of MC (curve a). The initial weight loss happened around 100 °C, which is due to the evaporation of physically adsorbed and hydrogen bond-linked water molecules [29]. A large rapid decrease of the weight occurred between 250 and 400 °C in the second stage. This could be assigned to the thermal degradation and decomposition of cellulose with the generation of C, CO, CO_2_ and H_2_O [30]. The thermal degradation behavior of MC_4_ aerogel (curve b) is the same as MC aerogel. The maximum decomposition temperature (T_max_) that occurs can be found in Figure 4B. There is no difference of T_max_ between MC and MC_4_ aerogels with T_max_ of around 360 °C. On the other hand, the total weight residue of MC_4_ aerogel is 5.74%, while MC aerogel has the total weight residue of 4.59%. The slightly higher total weight residue is due to the existence of amoxicillin, which confirms the successful loading of amoxicillin into the cellulose aerogel. 

### 2.4. Drug Loading and Drug Release

**Figure 5:**
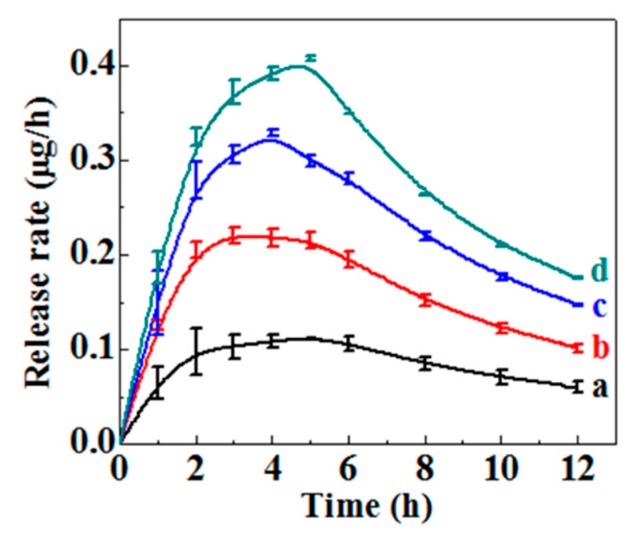
Amoxicillin release rates of MC_1_ (a), MC_2_ (b) MC_3_ (c) and MC_4_ (d) aerogels.

Amoxicillin loadings in cellulose aerogels were calculated and the result is shown in Table 1. It can be seen that the amoxicillin loading in the cellulose aerogel increases with the increase of initial amoxicillin concentration. The amoxicillin loading of MC_1_ aerogel with the lowest initial concentration of 0.3 g/L is 4.03 ± 0.07 μg/cm^2^. The loading of amoxicillin in the MC_4_ aerogel reaches 13.45 μg/cm^2^. In order to evaluate the release behavior of amoxicillin from cellulose aerogels, cumulative release behavior was monitored in PBS buffers at pH 7.4 and the calculated release rate of amoxicillin is displayed in Figure 5. As expected, the release rate of amoxicillin increases with the amoxicillin loading increasing in the cellulose aerogel. There is an initial rapid burst release for all tested aerogels in the first 2 h, which is thought to be associated with the drug that is absorbed or weakly bounded to the surface of cellulose aerogel [31]. Then, a gradually increased release rate of amoxicillin was shown. Eventually, a sustainable release lasting over 12 h was exhibited although the release rate began to decrease after 5 h. Thus, cellulose aerogel is proved to be a good amoxicillin carrier and it can control drug release behavior well. 

### 2.5. Antibacterial and Antifungal Performance

**Figure 6:**
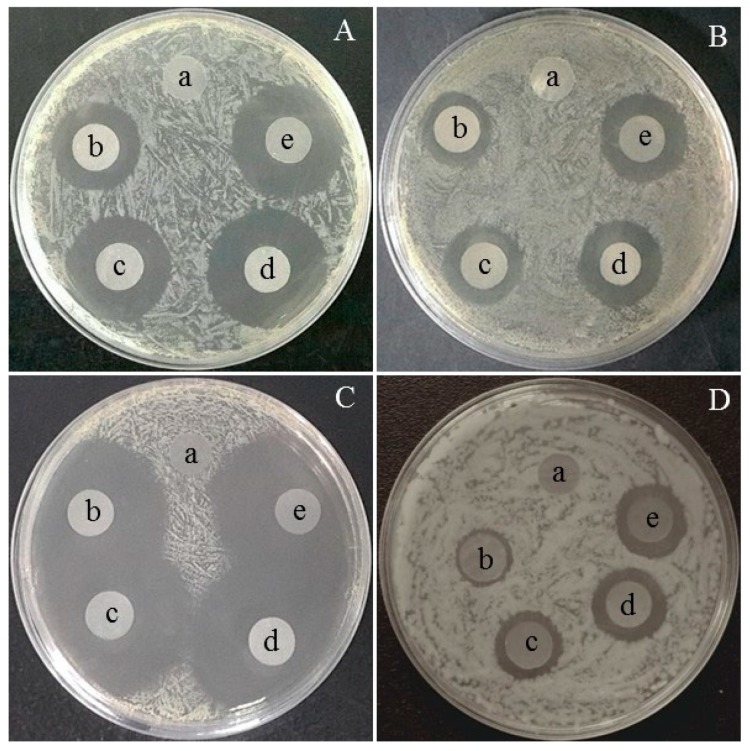
Optical images of inhibition zones of cellulose aerogels: (**A**) *E. coli*, (**B**) *C. albicans*, (**C**) *S*. *aureus* and (**D**) *B. subtilis*. (In all plates, a–e are MC, MC_1_, MC_2_, MC_3_ and MC_4_ aerogels).

The antibacterial and antifungal activities of prepared cellulose aerogels were studied by the disc diffusion method. The antibacterial capacity is determined by measuring the diameter of the clear zone of inhibition around the samples after 24 h incubation. The pictures are shown in Figure 6. As expected, no inhibition zones were observed for MC as control (a), implying that MC does not have any antibacterial or antifungal abilities. 

**Figure 7:**
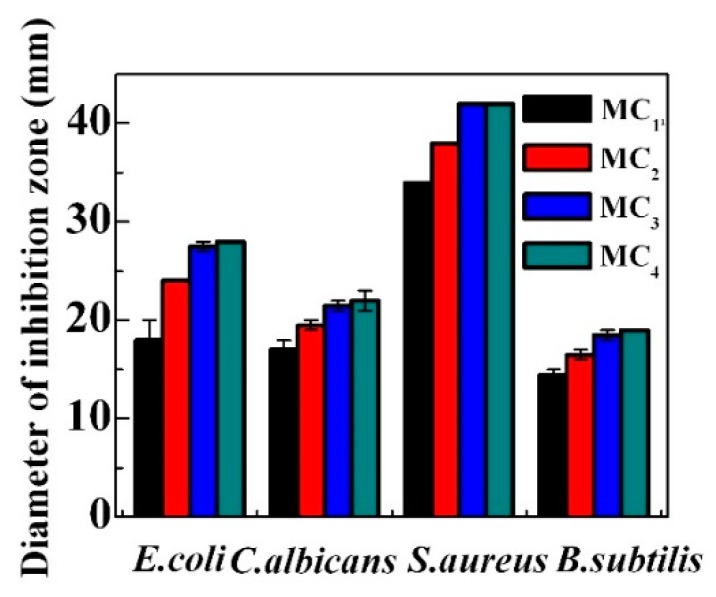
Average diameters of inhibition zones of cellulose aerogels, includes disk diameter of 10 mm.

The average diameters of inhibition zones of cellulose aerogels measured from the disc diffusion method are listed in Figure 7. It can be seen that the inhibition zone increases with the increase of amoxicillin loading in the cellulose aerogels. The trend levels out with the continuous increase of amoxicillin loading. No significant difference can be found between MC_3_ and MC_4_ aerogels. MC_4_ aerogel exhibits the best antibacterial activities and its zones of inhibition diameter of *E. coli*, *C. albicans*, *S. aureus* and *B. subtilis* are 28 mm, 22 mm, 42 mm and 19 mm, respectively. MC_1_ aerogel has the smallest diameters of inhibition zone of 18 mm, 17 mm, 34 mm and 15 mm against *E. coli*, *C. albicans*, *S. aureus* and *B. subtilis*. The present study clearly illustrates that the prepared cellulose aerogels show excellent antibacterial and antifungal activities.

## 3. Materials and Methods 

### 3.1. Preparation of Cellulose Aerogels

Cellulose aerogels were prepared by a facile solution casting method [32]. A transparent 5.8% cellulose solution was prepared by dissolving microcrystalline cellulose powder into 9% NaOH and then stirred at 4 °C for 2 h. The cellulose solution was casted into the template on glass plate, and soaked in a de-ionized water bath for 30 min, followed by rinsing with de-ionized water. Then, cellulose hydrogels were immersed into amoxicillin solutions with concentrations of 0.3, 0.6, 0.9 and 1.2 mg/mL for 24 h. The cellulose hydrogels were obtained by rinsing with de-ionized water, following by freeze-drying at −40 °C for 24 h. The final aerogels were named as MC_1_, MC_2_, MC_3_ and MC_4_, respectively. Then, cellulose aerogel without amoxicillin loading was labelled MC, which was used as the control. 

### 3.2. Characterization

A JSM-7600F SEM operating at an accelerating voltage of 10–15 kV was used to investigate the surface morphologies of cellulose aerogels. The samples were coated with a thin layer of platinum under high vacuum conditions (20 mA, 100 s). FTIR spectra were recorded on a Spectrum Two Spectrometer (Perkin Elmer, Akron, OH, USA) with the wavenumber range of 4000–400 cm^−1^ at a resolution of 4 cm^−1^. The grounded samples were prepared by mixing with dried KBr, and being pressed into a small tablet by compression. Thermogravimetric analysis (TG) was carried out by using a TA Instruments model Q5000 TGA. The samples were heated from 20 to 600 °C with a heating rate of 10 °C/min under nitrogen atmosphere.

### 3.3. Amoxicillin Loading Determination

Amoxicillin loadings were determined according to the original concentration (C_0_) and the unloaded concentration (C_1_) using a SHIMADZU UV 2450 spectrophotometer at the monitoring wavelength of 225 nm. Amoxicillin loading in the aerogels (L) was calculated with the following equation:

(1) $$\;L=\frac{\left(C_{0}-C_{1}\right)*V}{A}\;$$

where V is the total volume of original solution, and A is the area of cellulose aerogel.

### 3.4. In Vitro Release Assays

The aerogels were cut into round pieces in diameter of 10 mm. The release behaviors of amoxicillin were studied in PBS buffers [32]. At specific time points, an aliquot of 3.5 mL was collected from each solution and the absorbance was then measured at 225 nm by a SHIMADZU ultraviolet (UV) 2450 spectrophotometer. An equivalent volume of fresh PBS buffer was replaced into the system after each sampling to maintain constant medium volume. Thus, the cumulative released amounts can be calculated accordingly. The experiments were carried out in triplicate.

### 3.5. Antibacterial and Antifungal Activities

The antibacterial activities of cellulose aerogels were investigated by the disk diffusion method against *E. coli*, *C. albicans*, *S. aureus* and *B. subtilis*. Cellulose aerogels were cut into round shapes with 10 mm diameter and sterilized by ultraviolet lamp for 60 min. Lawns of test bacteria (about 1 × 10^5^ CFU/plate) were prepared on TSA. The sterilized aerogels were then carefully placed upon the lawns and placed in a 37 °C incubator for 24 h. Then inhibitory action of tested samples on the growth of the bacteria was determined by measuring the diameter of the inhibition zone.

## 4. Conclusions

Regenerated cellulose was fabricated by a facile green method and loaded with different concentrations of amoxicillin to obtain cellulose aerogels. Cellulose aerogels could release the drug in a sustained manner, displaying a long-lasting release after an initial burst release. The prepared cellulose aerogels display effective antibacterial activity against *E. coli*, *C. albicans*, *S. aureus* and *B. subtilis*. Therefore, the developed amoxicillin-loaded cellulose sponges have potential biomedical applications.

## Figures and Tables

**Table 1:** Amoxicillin loadings in cellulose aerogels.

	Initial Amoxicillin Concentration (mg/mL)	Amoxicillin Loading (μg/cm^2^)
MC_1_	0.3	4.03 ± 0.07
MC_2_	0.6	8.62 ± 0.55
MC_3_	0.9	11.85 ± 0.61
MC_4_	1.2	13.45 ± 0
